# Prevalence of Vitamin D Deficiency and Calcium Homeostasis in Saudi Children

**DOI:** 10.4274/jcrpe.3301

**Published:** 2016-12-01

**Authors:** Adnan M. Al Shaikh, Bahaa Abaalkhail, Ashraf Soliman, Ibrahim Kaddam, Khalid Aseri, Yousef Al Saleh, Ali Al Qarni, Ahmed Al Shuaibi, Waleed Al Tamimi, Abdel Moniem Mukhtar

**Affiliations:** 1 King Abdulaziz Medical City in Jeddah, King Saud bin Abdulaziz University for Health Sciences, Department of Pediatrics, Chemistry Laboratory, Community Medicine, Jeddah, Saudi Arabia; 2 King Abdulaziz University Hospital, Clinic of Family and Community Medicine, Jeddah, Saudi Arabia; 3 University of Alexandria Faculty of Medicine, Department of Pediatrics, Alexandria, Egypt; 4 King Abdulaziz Medical City-Central, King Saud bin Abdulaziz University for Health Sciences, Department of Medicine, Chmistry Laboratory, Riyadh, Saudi Arabia; 5 King Abdulaziz University Hospital, King Abdullah International Medical Research Center, Al Hasa, Saudi Arabia; 6 Imam Abdulrahman AlFaisal Hospital, Clinic of Family Medicine, Dammam, Saudi Arabia

**Keywords:** Vitamin D, Vitamin D deficiency, vitamin D insufficiency, parathyroid hormone levels, calcium, inorganic phosphate

## Abstract

**Objective:**

Vitamin D deficiency (VDD) and vitamin D insufficiency (VDI) are significant health problems all over the world. The aim of this study was to determine the prevalence of VDD and VDI in children and adolescents residing in 8 provinces in the Kingdom of Saudi Arabia and to also investigate calcium homeostasis in these subjects.

**Methods:**

A cross-sectional study was conducted in 2110 participants aged between 6 and 15 years. Information on socio-demographic status, anthropometric measurements, knowledge about vitamin D, color of the skin, dietary intake, sun exposure experience, smoking, and physical activity were collected through a questionnaire given to the parents of all subjects. The subjects were divided into three groups as vitamin D deficient, vitamin D insufficient, and vitamin sufficient according to their blood level of vitamin D [VDD ≤25 nmol/L (25 hydroxy vitamin D), VDI >25-50 nmol/L, and VDS >50 nmol/L].

**Results:**

VDD was highly prevalent in this group of children. 95.3 of the subjects had either VDD (45.5%) or VDI (49.9%). The prevalence rate of VDD combined with VDI was higher in females (97.8%) compared to males (92.8%) (p<0.001). Only 1.6% had significant hypocalcaemia. Children with dark skin had lower concentrations of vitamin D and higher concentrations of parathormone. A positive correlation was observed between 25 hydroxy vitamin D level and serum calcium, inorganic phosphate, and alkaline phosphatase concentrations.

**Conclusion:**

The results showed a high prevalence of VDD and VDI in Saudi children with significantly higher prevalence in girls. These findings necessitate the set-up of a national program for vitamin D supplementation and health education for this vulnerable group.

**WHAT IS ALREADY KNOWN ON THIS TOPIC?**

Vitamin D deficiency in children in Saudi Arabia was reported in previous small studies in single cities in the country.

**WHAT THIS STUDY ADDS?**

Herein, we present our large-scale study on prevalence of vitamin D deficiency and calcium homeostasis in Saudi children and adolescents covering almost the whole country.

## INTRODUCTION

Vitamin D is an important steroid hormone with endocrine, paracrine, and autocrine effects. It is produced endogenously in the skin by exposure to ultraviolet rays or can be taken from exogenous sources such as some food items and vitamin D preparations (1,2,3).

Vitamin D has a crucial role in enhancing physiological functions both in skeletal and extra-skeletal tissues. Its vitamin D deficiency (VDD) and vitamin D insufficiency (VDI) are associated with many acute and chronic illnesses including disorders of calcium (Ca) metabolism, autoimmune diseases, some cancers, type 2 and type 1 diabetes mellitus, cardiovascular disease, and infectious diseases (4,5,6).

Vitamin D is primarily synthesized in the skin after exposure to ultraviolet radiation (UVR) and less than 10% is derived from dietary sources. The quantity of vitamin D synthesized in the skin depends on the angle of the sun rays and thus on latitude, season, time of the day and duration of exposure. It is highest when the sun is in its zenith. However, sun exposure does not lead to any vitamin D3 production in the skin during most of the winter at latitudes above and below ~33 degrees North and South. Other factors influencing cutaneous vitamin D production adversely are increased skin pigmentation, aging, and the topical application of sunscreens (7,8). Food items which naturally contain vitamin D in significant amounts are very limited (9).

VDD is diagnosed when 25 hydroxy vitamin D [25(OH) vitamin D] is ≤25 nmol/L, while VDI is defined as a 25(OH) vitamin D level of >25-50 nmol/L. 25(OH) vitamin D >50 nmol/L is considered sufficient, with 75-150 nmol/L being the preferred range (10,11,12). The 2011 Institute of Medicine (IOM) committee, in agreement with the Lawson Wilkins Pediatric Endocrine Society, targeted a serum value for 25(OH) vitamin D of at least 50 nmol/L as meeting the needs of nearly all children as well as those of adults (13,14). Hypovitaminosis D is prevalent in the Middle East North Africa region (MENA) and in the Arab gulf countries (15). In these countries, a lack of population-based studies, as well as gaps in studies in infants, pre-pubertal children, and in adolescents hinder the development of region-specific guidelines and constitute a major obstacle to impact this chronic and most often subclinical disease (13,16,17,18).

The aim of this study was to determine the prevalence of VDD and VDI in a large cohort of Saudi children and adolescents in relation to their Ca homeostasis. Moreover, the study assessed Ca homeostasis parameters and factors associated with VDD, including age, skin color, and body mass index (BMI).

## METHODS

The study was approved by the King Abdullah International Medical Research Center (KAIMRC) and conducted during the years 2013-2014. It included 2110 apparently healthy male and female children (1013 male, 1097 female) aged 6-15 years. The subjects were recruited from primary, intermediate, and secondary schools of the Western, Central, and Eastern regions of Saudi Arabia. These schools represent all educational levels in the country. Due to limited resources, no participants from the Northern and the Southern regions of the country could be included in this survey.

Individuals with renal, liver, and gastrointestinal disease, as well as those on any form of drug treatment with possible effect on bone metabolism (e.g. corticosteroids, anticonvulsants, and/or thyroid hormones) were excluded.

Trained health workers helped in data collection and blood extraction. Parental consent was obtained ahead of time and covered all ethical issues related to the questionnaire and the study. All parents were given a questionnaire developed in Arabic language by the investigators and pretested and coded before the actual field work. The parents were also given a covering letter that explained the objectives of the study and included information (telephone number and emails) on the investigators for any inquires. The questionnaire included questions on socio-demographic state, anthropometric measurements, knowledge about vitamin D and VDD, skin color, dietary intake, sun exposure, smoking, and physical activity.

All participants underwent a general physical examination. Body weight to the nearest 0.1 kg was measured using a standard balance beam, and body height to the nearest 0.1 cm was measured using the Harpenden stadiometer (Holtain Ltd, Ales, UK). BMI was calculated as weight (kilograms) divided by height squared (square meters). Waist circumference was measured using anthropometric tape by determining the distance midway between the iliac crest and the lowest rib with the subject standing.

Blood samples were collected from the subjects in the 3 different regions throughout the academic year, which includes part of the summer and other seasons. Specimens were collected during the day, from 09:00 o’clock to 12:00 noon. In all samples, the serum was immediately separated and the samples were protected from light and stored at -70 °C. The samples were then sent in ice to a central laboratory (at King Abdulaziz Medical City in Jeddah). The specimens were analyzed in the central laboratory using the same method (chemiluminescence immunoassay) within 2 weeks of blood collection.

The cutoff points of the IOM for vitamin D levels, namely, ≤25 nmol/L for deficiency, >25-50 nmol/L for insufficiency, and >50 nmol/L for sufficiency were used in the analysis of the data (10-12). Intact parathyroid hormone (PTH), Ca, inorganic phosphate (PO_4_), alkaline phosphatase (ALP), and creatinine were determined in Architect machine (ABBOTT laboratories, Wiesbaden, Germany). Chemiluminescent Microparticle Immuno Assay (CMIA) was used for quantification of intact PTH in serum and plasma.

The sensitivity of the chemiluminescence immunoassay is <3.0 pg/mL and intra- and inter-assay CV percentages were 6.1% and 3.4% at a level of 69 pg/mL, respectively.

The outcome variable VDD was used as continuous variable and categorical variable and was divided into three categories according to the level of 25(OH) vitamin D, as given above.

### Statistical Analysis

To describe our study population, we used frequencies and absolute numbers for categorical variables and mean ± standard deviation values. Median and inter-quartile range values were used for continuous variables. Association between two categorical variables were assessed using the chi-squared test or the Fisher exact test when the data are sparse in one or more category. Associations between continuous variables were examined using either student’s t-test for unpaired samples or one-way analysis of variance (ANOVA), as appropriate. Linear regression equation was used to investigate possible relations between the different variables. For all statistical tests, a p-value of <0.05, two tail probability was accepted as significant. We used the Statistical Package for Social Sciences version 19 for data analysis.

## RESULTS

In this cross-sectional study of a large cohort of children of ages 6 and 15 years, the overall prevalence of combined VDD and VDI was 95.3% and that of vitamin D sufficiency only 4.7%. The prevalence in females (97.8%) was significantly higher than in males (92.8%). VDD [25(OH) vitamin D ≤25 nmol/L] was detected more frequently in females (63.9%) than in males (25.6%) (Table 1). VDD was more prevalent in the older age groups (47.2%) than in the younger groups (29.9%) as shown in Table 1.

As shown in Table 2, comparisons between two age groups (6-12 years versus 13-15 years) showed that the younger group had a higher mean 25(OH) vitamin D level (33.1±12.2 nmol/L) compared to the older group (27.6±11.4 nmol/L). Circulating PTH concentrations were significantly higher in the older group. Serum Ca and PO_4_ concentrations did not differ significantly between the two groups. None of the children in the young group had hypocalcemia defined as a serum Ca level of <2.1 nmol/L, while 0.8% of the older group had hypocalcemia.

Children with VDD (≤25 nmol/L) had significantly higher PTH concentrations and lower PO_4_ levels compared to those with higher 25(OH) vitamin D concentrations. Serum Ca levels did not differ between the two groups. Lower serum PO_4_ and higher PTH levels were observed in patients indicative of the presence of a compensatory response to low 25(OH) vitamin D levels (Table 3).

The degree of skin darkness of all children and adolescents was assessed and divided into 3 grades where 1=light (white), 2=brown, and 3 is black (Tables 4, 5). Comparison between the different groups according to their skin color revealed that children with black skin (group 3) had significantly lower 25(OH) vitamin D and higher PTH and ALP levels compared to those with lighter skin (ANOVA, p<0.001).

In this study, 47.1% of children and adolescents were not receiving any supplements of vitamin D, 16.5% were taking no milk or milk products, while 8.6% were taking milk less than once weekly. In 29% of the cohort, no exposure to the sun at any time was reported (Table 5) and 27% of the subjects were reported to wear complete covering clothes including the face and hands. In those who reported being exposed to the sun, 52% reported exposure at less efficient times for UV rays (early morning and late afternoon) and 10% reported use of sun-screening creams.

**Figure 1:**
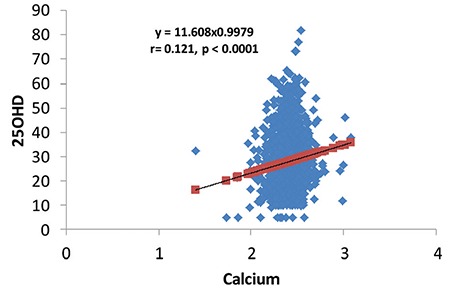
Regression of Vitamin D level on calcium concentration in population

**Figure 2:**
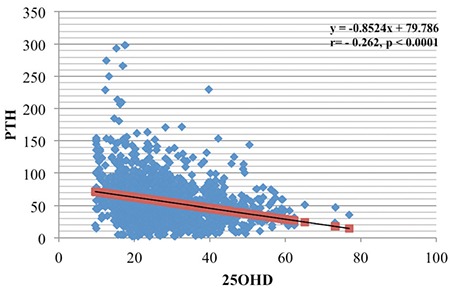
Regression of Vitamin D on parathyroid hormone level in population <15 years

**Figure 3:**
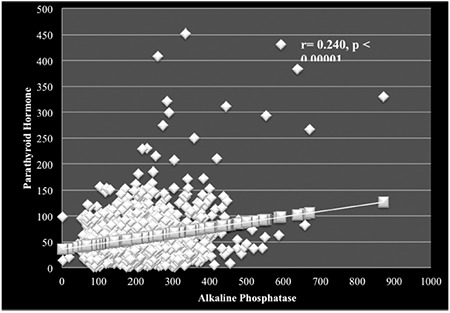
Regression of parathyroid hormone on alkaline phosphatase concentrations in population <15 years

Correlation analyses revealed that 25(OH) vitamin D levels correlated significantly with Ca level (r=0.15, p<0.001) (Figure 1) and negatively with PTH (Figure 2), age, and BMI. A positive correlation was observed between PTH and ALP levels (r=0.23, p<0.001) (Figure 3).

## DISCUSSION

Vitamin D is critical for Ca homeostasis and for mineralization of the skeleton, especially during periods of rapid growth, namely, growth in infancy, childhood, and pubertal period. Without vitamin D, only 10-15% of dietary Ca and about 60% of phosphorus are absorbed. The active form, 1,25-dihydroxy vitamin D [1,25-(OH)_2_D_3_] markedly increases the efficiency of intestinal Ca and phosphorus absorption. Serum levels of 25(OH) vitamin D below 50 nmol/L are associated with a significant decrease in intestinal Ca absorption. In children, adolescents, and adults, this is associated with increased PTH and decreased insulin-like growth factor 1 (IGF-1) secretion. Serum levels of 25(OH) vitamin D are directly related to bone mineral density with a maximum density achieved when the 25(OH) vitamin D level reaches 100 nmol/L (40 ng/mL) or more. Severe and/or prolonged VDD is associated with impaired linear growth and the development of many skeletal disorders including rickets, osteomalacia, and fractures. In addition, many extra-skeletal disorders have been associated with VDD. An increasing body of evidence also shows the extra-skeletal benefits of vitamin D, such as those on the immune system, fuel metabolism, cardiovascular system, and cancer. In addition, associations with decreased mortality have been reported (13,17,18,19,20,21,22,23,24,25,26,27,28,29).

The results of this study showed a very high prevalence of VDD and VDI in the majority of children and adolescents between 6 and 15 years of age in a sunny country such as the Kingdom of Saudi Arabia. Several large-sample populations-based studies as well as smaller studies also revealed a high prevalence of hypovitaminosis D and of rickets in infants (0.5% of Saudis <2 years) and adolescents in Saudi Arabia (21,22,23,24,25,30).

While rickets is almost eradicated in western populations, its prevalence remains unacceptably high in Asia, Africa, and the Middle East and resurgence is also registered in ethnic minority groups in some Northern European countries despite its plentiful sunshine. Such findings are explained by the prevalence of specific risk factors for hypovitaminosis D in this region. These include lifestyle factors, namely lack of sunlight exposure (because of very hot weather) (29% of our cohort) or exposure during the wrong time of the day (52% of our cohort) and inadequate use of vitamin D supplements (47% of our cohort) are well recognized major determinants of circulating 25(OH) Vit D levels.

Many studies reported significant lack of sun exposure and lack of vitamin D supplementation in the Arab Gulf region (26,27,28,29,31,32). A study conducted in Riyadh city which included 808 Saudi children and 561 adults of both genders showed that subjects who had a sun exposure of <20 min and who were of dark skin had the highest prevalence of VDD (33). In our study, data on duration of sun exposure, time of exposure, clothing, using sun protection substances were also collected. These factors working independently or interacting with each other will be analyzed in reference to skin color in future papers.

It has been reported that genetic factors may contribute to up to 50% of inter-individual variability in serum 25(OH) vitamin D levels and in 27% of Saudi patients (32). The study done by Baroncelli et al (34) on 98 rachitic children conducted in Egypt and Turkey showed that VDD and rickets in the Middle East countries was determined, in addition to nutritional and environmental factors, also by genetic predisposition factors. They found that vitamin D receptor genotypes may predispose to rickets by increased frequency of the F allele.

In our study and also in other studies, females had more prevalent VDD compared to males. In adolescents and adults, this finding may be due to the more restricted outdoors activity of the females and the conservative clothing they wear that prevent exposure to the sun rays. In addition, lack of awareness of the importance of sun exposure for bone health and for cosmetic reasons (avoiding darkening of their skin) including using sun-screening agents are other important factors.

A very recent study in children and adolescents from Iran to determine the reference intervals for vitamin D and other 12 biochemical indices showed the cutoff level for VDD to be 50 nmol/L (35). We also used this cutoff point in our study and found a high prevalence of VDD in our population despite the sunshine all the year around, a finding which can be associated with the influence of other interacting risk factors that need to be investigated in further studies.

The majority of the children with VDD in our cohort, even those with severe VDD [25(OH) vitamin D <25 nmol/L], had normal serum Ca, PO_4_, and ALP levels. The development of clinical manifestations of VDD rickets and osteomalacia depends on many factors apart from the severity and duration of the VDD. A potent adaptation process, mediated by the PTH and the IGF-1 modifies the clinical and radiological manifestations of VDD (12,13). Therefore, overt cases of rickets and osteomalacia represent only the tip of the iceberg of patients with severe VDD and may indicate a defect in adaptation. It is also noteworthy that when clinical rickets develops, the entire process occurs rapidly, within a few months. This potent adaptation process, brought about by increased PTH secretion, explains the normal Ca level of our black children and adolescents despite their significantly low 25(OH) vitamin D levels. In addition, this same adaptation process explains the relatively low incidence of florid rickets and osteomalacia in the presence of a high prevalence of VDD. In support of this view, the children in our cohort with 25(OH) vitamin D <25 nmol/L values have significantly higher PTH and lower PO_4_ levels (due to the phosphaturic effect of PTH), to maintain their Ca levels within the normal range. Consequently, VDD in adolescents may be asymptomatic and go undetected. These patients usually present with vague manifestations including pain in weight-bearing joints (back, thighs, calves), difficulty in walking, running and/or climbing stairs, getting up from a squatting position, and muscle cramps. Facial twitches and carpo-pedal spasms are less frequent symptoms. Due to the demineralization of bones, deformities such as triradiate pelvis, lordosis, and/or genu valgus or varus may develop. These manifestations may go unnoticed for long periods. In severe and prolonged deficiency, vertebral compression fractures and fractures of the long bones may occur. Moreover, VDD can be misdiagnosed as fibromyalgia, chronic fatigue syndrome, or simply depression in adolescents (13,36,37).

In conclusion, VDD is highly prevalent in Saudi children, both in females and males. Most of the cases are asymptomatic or may present with vague and non-specific symptoms. These data urge pediatricians and physicians to have a higher degree of clinical suspicion for VDD and to screen all the patients with non-specific musculoskeletal pain by measuring 25(OH) vitamin D level. Food fortification with vitamin D and health education in schools and media to improve sun exposure appear to be very important steps to correct this prevalent deficiency and prevent its short- and long-term deleterious consequences. In addition, regular screening of children for VDD and initiation of treatment at an early stage are important measures for prevention of the undesirable consequences of VDD.

## Ethics

Ethics Committee Approval: King Abdullah International Medical Research Center (KAIMRC), Jeddah, Saudi Arabia 2012-2013, Informed Consent: It was taken.

Peer-review: Internally peer-reviewed.

## Figures and Tables

**Table 1:**
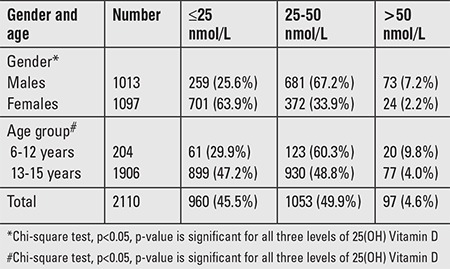
Prevalence of vitamin D deficiency and vitamin D insufficiency in the study group by gender and age group

**Table 2:**
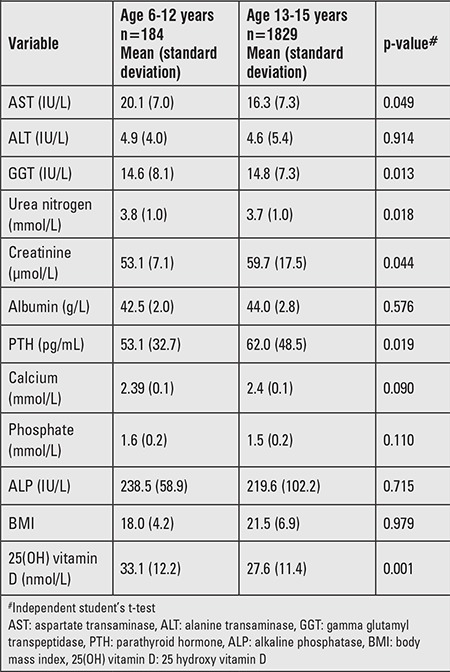
Relationship between age groups and selected variables of calcium homeostasis

**Table 3:**
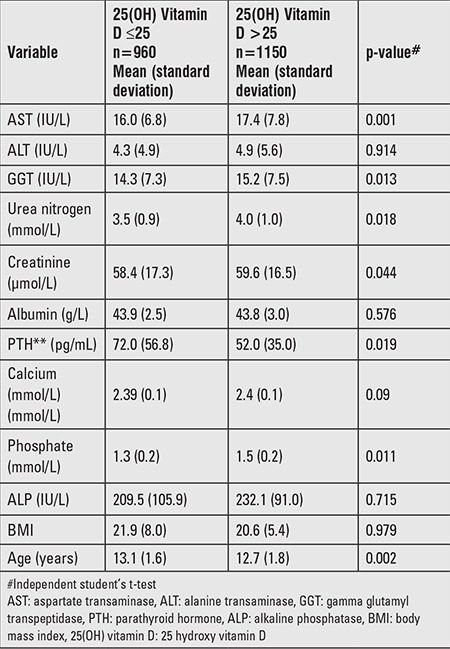
Calcium homeostasis in children according to vitamin D level

**Table 4:**
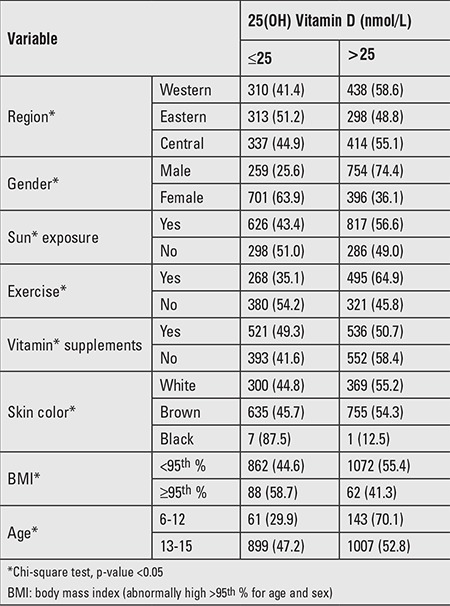
Prevalence of vitamin D deficiency according to age, gender, region, skin color, and exposure to the sun

**Table 5:**
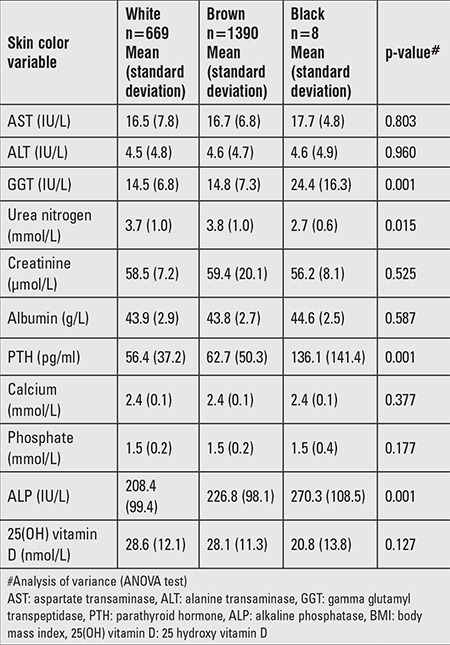
Relationship between vitamin D and other biochemical data according to skin color
